# Kinematic and dynamic gait compensations resulting from knee instability in a rat model of osteoarthritis

**DOI:** 10.1186/ar3801

**Published:** 2012-04-17

**Authors:** Kyle D Allen, Brian A Mata, Mostafa A Gabr, Janet L Huebner, Samuel B Adams, Virginia B Kraus, Daniel O Schmitt, Lori A Setton

**Affiliations:** 1 J. Crayton Pruitt Family Department of Biomedical Engineering, University of Florida, Gainesville, FL, USA; 2 Department of Biomedical Engineering, Duke University, Durham, NC, USA; 3 Department of Orthopaedic Surgery, Duke University Medical Center, Duke University, Durham, NC, USA; 4 Department of Medicine, Division of Rheumatology, Duke University Medical Center, Duke University, Durham, NC, USA; 5 Department of Evolutionary Anthropology, Duke University, Durham, NC, USA

## Abstract

**Introduction:**

Osteoarthritis (OA) results in pain and disability; however, preclinical OA models often focus on joint-level changes. Gait analysis is one method used to evaluate both preclinical OA models and OA patients. The objective of this study is to describe spatiotemporal and ground reaction force changes in a rat medial meniscus transection (MMT) model of knee OA and to compare these gait measures with assays of weight bearing and tactile allodynia.

**Methods:**

Sixteen rats were used in the study. The medial collateral ligament (MCL) was transected in twelve Lewis rats (male, 200 to 250 g); in six rats, the medial meniscus was transected, and the remaining six rats served as sham controls. The remaining four rats served as naïve controls. Gait, weight-bearing as measured by an incapacitance meter, and tactile allodynia were assessed on postoperative days 9 to 24. On day 28, knee joints were collected for histology. Cytokine concentrations in the serum were assessed with a 10-plex cytokine panel.

**Results:**

Weight bearing was not affected by sham or MMT surgery; however, the MMT group had decreased mechanical paw-withdrawal thresholds in the operated limb relative to the contralateral limb (*P *= 0.017). The gait of the MMT group became increasingly asymmetric from postoperative days 9 to 24 (*P *= 0.020); moreover, MMT animals tended to spend more time on their contralateral limb than their operated limb while walking (*P *< 0.1). Ground reaction forces confirmed temporal shifts in symmetry and stance time, as the MMT group had lower vertical and propulsive ground reaction forces in their operated limb relative to the contralateral limb, naïve, and sham controls (*P *< 0.05). Levels of interleukin 6 in the MMT group tended to be higher than naïve controls (*P *= 0.072). Histology confirmed increased cartilage damage in the MMT group, consistent with OA initiation. *Post hoc *analysis revealed that gait symmetry, stance time imbalance, peak propulsive force, and serum interleukin 6 concentrations had significant correlations to the severity of cartilage lesion formation.

**Conclusion:**

These data indicate significant gait compensations were present in the MMT group relative to medial collateral ligament (MCL) injury (sham) alone and naïve controls. Moreover, these data suggest that gait compensations are likely driven by meniscal instability and/or cartilage damage, and not by MCL injury alone.

## Introduction

Osteoarthritis (OA) results in articular cartilage loss, bone remodeling, and upregulation of proinflammatory and catabolic mediators within the joint [1]. Despite a complex and indistinct etiology, OA ultimately leads to pain, joint dysfunction, and decreased patient quality of life. In contrast to OA in humans that is typically assessed through self-reports of pain and disability [2-5], assessment of preclinical OA models tends to focus on histological changes of the joint [6]. This disconnect may appear minor, but it is a significant challenge in the preclinical-to-clinical translation of OA therapeutics and diagnostics. Whereas OA degeneration and symptoms coincide, the severity of pain and disability does not necessarily correlate to the scale of degeneration [7-9].

Rodent behaviors can be used to describe some of the symptomatic consequences of OA in the preclinical OA model. Weight distribution during an induced-rearing posture has demonstrated weight shifts to the contralateral limb after the induction of OA [10-12]. Moreover, increased sensitivity to non-noxious mechanical stimuli, a condition known as tactile allodynia, has been observed in the OA-affected limb of preclinical OA models [11,12]. Although these behavioral assays are useful, a more complete behavioral analysis of rodent OA models would provide a clearer picture of the relationships between disease parameters and disease sequelae.

Gait assessment provides a tool to evaluate the consequences of OA in both the preclinical OA model and the OA patient. Patients with either hip or knee OA demonstrate gait asymmetries, in which both stance time and peak vertical force are reduced on the OA-affected limb; moreover, these quantitative measures align well with self-evaluation [13-17]. Similar tools now exist to assess rodent gait characteristics [18-25]; thus, spatiotemporal evaluations of gait patterns (stance time balance and gait symmetry) and dynamic assessments of three-component group reaction force can be evaluated in both the preclinical OA model and the OA patient. Moreover, a better understanding of gait compensations in combination with biologic markers of disease in rodent OA models will allow a better understanding of how disease parameters associate with disease sequelae.

History of ligament or meniscus injury is one factor associated with significant risk of premature OA development [26,27]. After traumatic knee injury, an upregulation of proinflammatory and catabolic mediators occurs in combination with changes in joint loading and chondrocyte metabolism. These changes initiate a chronic cycle that ultimately destroys a joint's articular surface and leads to the symptomatic presentation of OA [28-30]. Knee instability injuries can be simulated in preclinical rodent models by surgical destabilization of the joint through either anterior cruciate ligament (ACL) transection or medial collateral ligament (MCL) and medial meniscus transection (MMT). These instabilities lead to the development of cartilage lesions and bone remodeling [6,12,31,32]. In rodent knee instability models, studies have shown that a period of 4 to 6 weeks is sufficient to create severe cartilage lesions that simulate late-stage OA [6,12,31-34]; thus, a relatively rapid disease development occurs that is favorable for preclinical studies.

The objective of this study was to evaluate changes in rodent gait in a rat knee instability model. We hypothesize that rats with medial meniscus transection will develop a limp, spend less time on their injured limb relative to their contralateral limb, and distribute load unequally between the hindlimbs. In addition, the ability of gait assessments to describe the behavioral consequences of OA is compared with established behavioral assessments of tactile allodynia and incapacitance meter-based weight distribution. Finally, end-stage behaviors are correlated to cytokine concentrations in the serum and histopathology scores for the knee joint, investigating correlates between OA-related behaviors, biomarkers, and joint-level remodeling. Our results demonstrate that measures of rodent gait can evaluate the progression of pain and disability in the preclinical rodent OA model and may correlate well to joint-level remodeling and markers of OA pathogenesis.

## Methods

### Animal surgery and experimental timeline

Sixteen Lewis rats (male, 200 to 250 g) were acquired from Charles River Laboratory for the study. Animals were acclimated to the housing facilities at Duke University for 1 week before surgery. On day 0, 12 animals were anesthetized by using pentobarbital (60 mg/kg intraperitoneally) with anesthesia maintained with 2% isoflurane inhalation. The MCL of the right knee was exposed through a medial midline skin incision, and the MCL was transected. Six animals received only MCL transection (MCL sham). In the remaining six animals, the central portion of the medial meniscus was transected to simulate a complete radial tear (medial meniscus transection, MMT). Skin incisions were closed with 5-0 Vicryl sutures, and animals were allowed to recover from anesthesia. Sutures were removed at 7 days. A separate set of animals matched for age and strain that did not undergo any surgical procedure served as "naïve controls" (*n *= 4).

Naïve, MCL sham, and MMT animals were followed for 28 days. Tactile allodynia and spatiotemporal gait characteristics were assessed on postoperative days 9, 16, and 23; weight-bearing and dynamic gait characteristics were assessed on postoperative days 10, 17, and 24. On day 28, animals were euthanized by exsanguination under deep anesthesia (pentobarbital, 90 mg/kg intraperitoneally). Blood collected via cardiac puncture (1-ml syringe with 27-gauge × 1-inch needle) was spun in a separating Vacutainer (3,500 g, 15 minutes; BD Vacutainer, Franklin Lakes, NJ, USA), and the serum component was stored at -80°C. Operated and contralateral knee synovial fluid was collected with a 100-μl lavage. Here, the patellar ligament was transected and retracted with forceps. Saline was injected into and retrieved from the femoral groove; this process was repeated 3 times with the same saline to mix the saline with the synovial fluid. The final collected volume was transferred to an Eppendorf tube, spun to remove cellular constituents, and stored at -80°C. Knees were then dissected, removing excess skin and muscle tissue, fixed in 10% formalin for 48 hours, decalcified for 1 week in Cal-Ex decalcifying agent (Fisher Scientific, Fair Lawn, NJ, USA), and then paraffin embedded by using standard histology practices. All procedures described herein were approved by the Duke University Institutional Animal Care and Use Committee.

### Tactile allodynia

Mechanical paw withdrawal thresholds were determined by using an up-down protocol for von Frey filaments [35]. In brief, rats were acclimated to a wire-bottom cage for 30 minutes. Von Frey filaments (Stoelting, Wood Dale, IL, USA) were then applied to the plantar surface of the rats' hindpaws. If withdrawal occurred, the next-smallest filament was applied; if withdrawal did not occur, the next-largest filament was applied. With this method, we approximated the mechanical force in which paw withdrawal and stimulus tolerance are equally likely (paw withdrawal threshold) [35]. A full-factorial analysis of variance (ANOVA) with two factors (Surgical group, Time) was used to assess differences in the operated limb, with a *post hoc *Tukey HSD test to identify surgical group differences when indicated. In addition, differences between the operated and contralateral limb within the MCL sham and MMT groups were assessed by using a *t *test corrected for multiple comparisons.

### Spatiotemporal gait characterization

To assess spatiotemporal characteristics of rodent gait, rats were placed in an open arena (5 feet 6 inches × 1 foot 6 inches) with a glass floor, three transparent acrylic sides, black acrylic back and lid, and mirror oriented at 45 degrees underneath the arena floor. This arena allows simultaneous viewing of foot placements in the sagittal and ventral planes. As a rat passes through the middle 4 feet of the arena, a high-speed videocamera is manually triggered to capture the animal's movement (Phantom V4.2, 200 frames per second; Vision Research, Wayne, NJ, USA). Rats were allowed to explore the arena freely until five acceptable videos were acquired (10 to 25 minutes per animal); all recorded trials contained two to five complete gait cycles with less than 15% velocity change. Calibration grids of 1 × 1 cm were used to convert video pixels to geometric coordinates. Trial velocity was analyzed by using a custom MATLAB code wherein a background image was subtracted from each video frame; the subtracted frame was converted to a binomial image (threshold equal to 25% of the graythresh function return; MATLAB), and the centroid of the animal was calculated for all frames (regionprops; MATLAB). Velocity and direction of travel were subsequently calculated from these positional data. The position of the hindpaws and frame of foot-strike and toe-off events were determined through manual digitization [19,20,36,37].

Pixel coordinates of the hindpaw position and frame numbers were converted into geometric and time variables, and the following spatiotemporal characteristics were calculated: stride length, step width, percentage stance time, and gait symmetry. Percentage stance time, also known as limb duty factor [38], is a limb's stance time divided by stride time. Percentage stance time tends to be balanced between the left and right limbs of a limb pair; or mathematically, the left hindlimb percentage stance time minus right hindlimb percentage stance time is near zero. This comparison of stance times (termed percentage stance time balance) can indicate that a gait abnormality and potential unilateral injury may exist [18,19,37]. Gait symmetry measures the uniformity of the foot-strike events in time. During walking, rodent gait is typically symmetric; or mathematically, the time between left and right foot-strikes is approximately half of time between two left foot-strikes [39]. If this variable (termed gait symmetry) is significantly different from 0.5, the foot-strike sequence is syncopated, indicating an asymmetric gait pattern, and a potential unilateral injury may exist [18,19,37].

For this statistical analysis, we compared each group at each time point with the mathematical definitions for balanced, symmetric gait by using a repeated-measures *t *test, with a statistically significant shift indicating an imbalanced and/or asymmetric gait pattern. A full-factorial two-factor ANOVA (Surgical group, Time) was used to assess statistical significance between treatment groups for velocity, percentage stance time balance, and symmetry, with a *post hoc *Tukey HSD test to identify treatment differences when indicated. Step width and stride length have mild to strong correlations with an animal's selected velocity; to account for this covariate, a generalized linear model (GLM) with two categorical factors (Surgical group, Time) and a linear dependence on trial velocity was used to assess statistical significance between treatment groups, with a *post hoc *Tukey HSD test to identify surgical group differences when indicated.

### Weight distribution

Hindlimb weight distribution was measured on an incapacitance meter (IITC, Inc., Woodland Hills, CA, USA), a behavioral analysis assay that measures weight bearing on the hindlimbs while the animal is in an induced rearing posture [10]. In brief, an incapacitance meter consists of two scales and specialized caging to encourage a rearing posture. Weight on the left and right hindlimbs was acquired during 5-second intervals (five trials per rat). These data were converted into weight distribution by dividing the weight on the right limb by the total weight for both hindlimbs. Weight-distribution imbalance was determined at each time point by using a repeated-measures *t *test, with balanced weight distribution represented by a right limb percentage weight near 50%. A full-factorial two-factor ANOVA (Surgical group, Time) was used to assess statistical significance, with a *post hoc *Tukey HSD test to identify surgical group differences when indicated.

### Dynamic gait characterization

To assess gait dynamics, rats were placed in a custom-built force plate arena (4 feet 6 inches × 6 inches) composed of three acrylic sides, a black acrylic side (back of the arena), and a medium-density fiberboard floor. At its center, a 1-inch × 6-inch section of the floor was isolated and attached to an overload-protected portable Hall-effect-based force plate (6 × 6 × 1.16 inches; ± 2.45 N x- and y-axis, +4.9 N z-axis, 200 Hz; Advanced Mechanical Technology, Inc., Watertown, MA, USA), calibrated as previously described [25]. Rats explored the force-plate arena for a 25-minute period; when the rat's hindlimb struck the isolated section of the floor, ground reaction forces were collected. Trials in which a portion of the foot landed on the isolated section of floor were excluded from the analysis (*post hoc *exclusion via video replay of each trial; Sony Handycam). Trials were obtained on both limbs for all animals; however, the number of trials for each animal was inconsistent between groups, limb, or time-point because of exclusions and an inability to control which hindlimb contacted the plate (unbalanced repeated measures).

**Figure 1:**
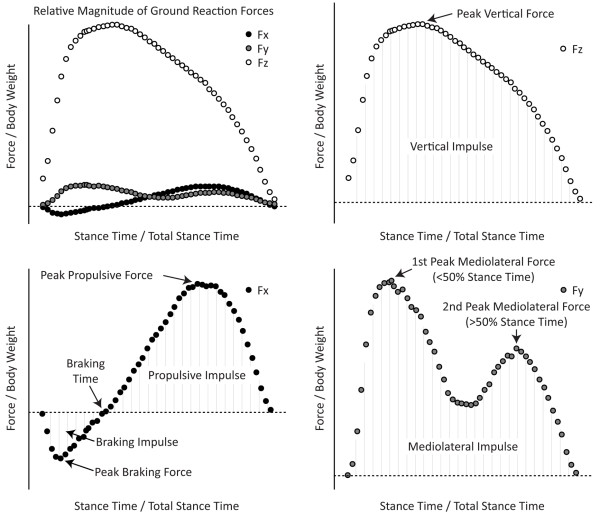
**Generalized measurement to describe the ground reaction force curves of a walking rat**. Ground reaction forces were reduced into generalized measurements, as described by Howard and co-workers [24]. The relative size of the ground reaction forces are described in the top left panel. Vertical (Fz) forces are the largest ground reaction forces; here, a loading, support, and unloading phase can be observed (open circles, top right). Vertical ground reaction forces were generalized into peak vertical force (the largest vertical force achieved) and vertical impulse (the area under the Fz-time curve). Braking-propulsion forces (Fx) resemble a negative sine wave (solid circle, bottom left). Braking forces (-Fx) slow the linear translation of the center of mass in the direction of travel. These forces are observed early within the Fx-time curve and can be generalized into peak braking force and braking impulse. Propulsive forces (+Fx) propel the center of mass along the direction of travel. These forces occur later in Fx-time curve and can be generalized into peak propulsive force and propulsive impulse. Finally, mediolateral forces are directed at the midline of the animal and tend to have two distinct peaks (shaded circles, bottom right). The first peak (at < 50% stance time) represents the slowing of the center of mass as it is being transferred from the contralateral limb to the ipsilateral limb; or in other words, the center of mass is being translated onto the ipsilateral limb such that the contralateral limb may enter its swing phase. This region is generalized as the first peak mediolateral force. The second peak (at > 50% stance time) represents the force that must be generated to propel the center of mass to the contralateral limb such that the ipsilateral limb may enter its swing phase. This region is generalized as the second peak mediolateral force. The mediolateral force curve can also be generalized by the mediolateral impulse.

Force-plate data were imported into MATLAB, passed through a 25-Hz low-pass filter, and normalized to body weight and stance time. This normalization allows ground reaction forces to be described by dimensionless terms that are less sensitive to differences in an animal weight and changes in stance time between trials. Coordinate axes were defined such that +Fx indicated propulsive forces in the direction of travel (with -Fx indicating braking forces), +Fy indicated mediolateral forces directed toward the animal's midline for both hindlimbs, and +Fz indicated vertical force perpendicular to the contact area. Generalized terms representing the shape of each curve [24] were used for statistical analysis, as follows: Fx ground reaction forces were described by peak braking force, peak propulsive force, braking phase impulse, and propulsive phase impulse. Fy ground reaction forces were described by 1^st ^peak mediolateral force, 2^nd ^peak mediolateral force, and mediolateral impulse. Fz ground reaction forces were generalized by peak vertical force and vertical impulse (Figure 1). A full-factorial two-factor ANOVA was used to assess statistical significance for the operated limb, with a *post hoc *Tukey HSD test to identify surgical group differences when indicated. In addition, operated and contralateral limb differences were assessed by using a two-factor ANOVA (Limb, Time) within the MCL sham and MMT groups.

#### Serum/synovial fluid analysis

Synovial fluid samples were lyophilized and reconstituted to a volume to 50 μl. The concentrations of urea in 3-μl samples of sera and joint fluid were determined to correct for the dilution in synovial fluid induced by lavage [40]. Quantification of urea in this manner is accomplished by using enzyme reaction reagents that depend on the rate of utilization of nicotinamide adenine dinucleotide (NAD) and can be measured photometrically at 365 nm in small volumes by using by a CMA600 microdialysis analyzer (CMA Microdialysis, Solna, Sweden). Cytokines and chemokines in sera and synovial fluid were measured by using a Rat Cytokine 10-Plex Panel to quantify interferon gamma (IFN-γ), interleukin-1 alpha (IL-1α), IL-1β, IL-2, IL-4, IL-6, IL-10, IL-12 (p40/p70), granulocyte macrophage colony-stimulating factor (GM-CSF), and tumor necrosis factor alpha (TNF-α) (Invitrogen, Carlsbad, CA, USA). All samples were analyzed as recommended by the manufacturer by using a standard range of 0 to 3,200 pg/ml and a total of 50 μl of sample. The raw cytokine data were not normally distributed (Shapiro-Wilk *W *test < 0.05); thus, a log transformation was conducted to transform the cytokine concentrations into normally distributed data before assessing cytokine differences. A one-factor ANOVA (Surgical group) was conducted on log-transformed cytokine concentrations, with a *post hoc *Tukey HSD test to identify surgical group differences when indicated.

### Histology

Serial coronal plane knee sections (8 μm) were acquired for each animal. A single slide representing the most severe lesion formation on the medial tibial plateau was selected for toluidine blue staining (three to four sections per slide). Pathologic changes were graded by two blinded reviewers coming to consensus by using the OARSI Osteoarthritis Histopathology Assessment System [41]. This system assigns one of seven grades to a section based on evidence of progressive cartilage and subchondral bone damage encompassing normal cartilage, chondrocyte cell death, fibrillation, fissures, cartilage erosion, denudation, and osteophyte formation with subchondral bone remodeling (grade 0, cartilage intact; grade 6, deformation and evidence of bone remodeling). Kruskal-Wallis median tests were used to detect differences in OARSI grades between groups. By using the updated recommendations for the OARSI Osteoarthritis Histopathology Assessment System [42], measures of the lesion depth, width, and volume were assessed by using ImageJ [43]. These data were assessed by using a one-factor ANOVA (Surgical group), with a *post hoc *Tukey HSD test to identify surgical group differences when indicated.

### Correlation analysis

After ANOVA and GLM investigations, a subset of outcome measures of animal behavior or serum cytokine concentrations with evidence of significant (*P *< 0.05) or near-significant (0.05 ≤ *P *< 0.10) differences among groups was selected for study of potential correlations with measures of cartilage lesion formation. Correlations were assessed by using a univariate linear regression model of Y = β_0 _+ β_1 _X, where Y is a measure of cartilage lesion formation, × is an outcome measure of animal behavior or serum cytokine levels, β_0 _is an intercept term, and β_1 _is a slope term describing the association between the dependent variable Y and the independent variable X. A significant linear correlation is identified by β_1 _differing from zero at a significance of 0.05.

## Results

### Weight and weight distribution

**Figure 2:**
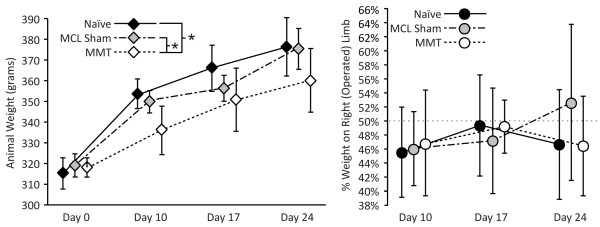
**Weight and weight distribution**. During the course of the experiment, weight increased in all treatment groups (left); however, rats with medial meniscus transection (MMT) surgery (open diamond) added less weight over time than naïve control animals (solid diamond) and rats with medial collateral ligament (MCL) sham surgery (shaded diamond, *P *= 0.003, *P *= 0.011, respectively). Imbalanced weight distributions were not observed in any group at any time (right: naïve control, closed circle; MCL sham, shaded circle; MMT surgery, open circle). Data are presented as mean ± SEM. *Differences between surgical groups at a level of *P *< 0.05.

During the experiment, weight increased in all animals; however, the weight of MMT rats increased more slowly than that of MCL sham or naïve animals (Figure 2, left; *P *= 0.011, *P *= 0.003, respectively). Imbalanced weight distributions, as measured with the incapacitance meter, were not observed in any group at any time (Figure 2, right), nor were differences identified among groups.

### Tactile allodynia

**Figure 3:**
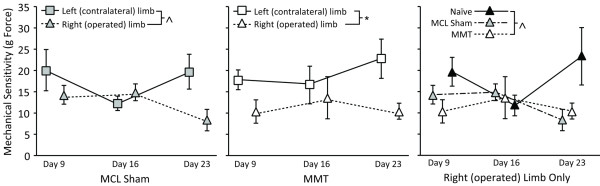
**Tactile allodynia**. Rats with medial meniscus transection (MMT) surgery had lower right (operated) paw withdrawal thresholds (open box) than their contralateral limb (open triangle; *P *= 0.017, middle) and tended to have lower right (operated) paw withdrawal thresholds than naïve controls (solid triangle, *P *= 0.056, right), but not medial collateral ligament (MCL) sham animals (shaded triangle). Rats with MCL sham surgery also tended to have differences between the operated limb (shaded triangle) and the contralateral limb (shaded box, *P *= 0.065, left). Data are presented as mean ± SEM. *Differences between surgical groups at a level of *P *< 0.05; ^differences near significance at a level of 0.1 <*P *≤ 0.05.

MMT rats also had lower paw withdrawal thresholds in their operated limb relative to their contralateral limb (*P *= 0.017), with MCL sham animals tending to have differences between the operated and contralateral limb (*P *= 0.065; Figure 3). MMT rats tended to have lower paw withdrawal thresholds in their operated limb than did naïve controls (*P *= 0.056), but were not different from MCL-sham animals (*P *= 0.914).

### Spatiotemporal gait characteristics

**Figure 4:**
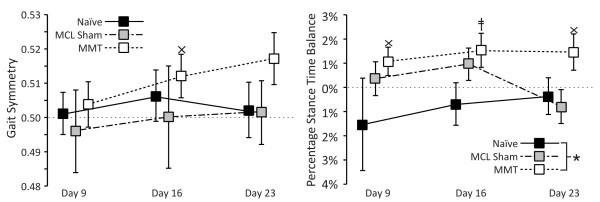
**Temporal gait pattern**. At day 9, gait symmetry was near 0.5 for all groups, indicating that the gait pattern was symmetric. However, with time, the gait pattern of the medial meniscus transection (MMT) group became progressively asymmetric (open box, left). At day 16, the gait pattern of MMT rats was near asymmetric (×; *P *= 0.089); by day 23, the gait of MMT rats was significantly different from that of symmetric gait rats (ǂ, symmetry ≠ 0.5; *P *= 0.020). Although naïve controls (solid box) and medial collateral ligament (MCL) sham animals (shaded box) had reasonably equivalent stance time on the left and right hindlimbs, MMT rats tended to have imbalanced stance times (balance ≠ 0.0, right). Although not significant for each time point, a general trend for MMT rats to spend less time on their operated limb relative to the contralateral limb can be observed (*P *= 0.077, 0.039, and 0.061 at days 9, 16, and 23, respectively). Data are presented as mean ± SEM. ǂValues that are significantly different from the mathematical definition for symmetric, balanced gait at a level of *P *< 0.05; ×values near significance at a level of 0.1 <*P *≤ 0.05. *Significant differences between surgical groups at a level of *P *< 0.05.

Gait became progressively asymmetric in the MMT group with time (Figure 4, left). By day 16, the gait of MMT rats tended to be asymmetric (*P *= 0.089); by day 23, the gait of MMT rats was significantly different from a symmetric gait pattern (*P *= 0.020). The gait of MMT rats also tended to be imbalanced (Figure 4, right), indicating that MMT rats spent less time on their operated limb than on their contralateral limb while walking (*P *= 0.077, 0.039, 0.061, at days 9, 16, and 23, respectively). The stance time balance of MMT rats was significantly different from that of naïve controls (*P *= 0.019).

The stance time of rats ranged from 0.16 to 0.80 seconds (average, 0.37 seconds); thus, changes observed in the temporal gait pattern of MMT rats are on the order of 0.001 to 0.025 seconds. It is noteworthy that these temporal changes are not detectable with the naked eye and require the use of high-speed videography.

With time, velocity increased (*P *= 0.015; 38.2 ± 9.5 cm/sec, 41.0 ± 10.1 cm/sec, and 44.8 ± 12.1 cm/sec on days 9, 16, and 23, respectively); stride length increased (*P *< 0.001; 14.2 ± 1.4 cm, 15.1 ± 1.6 cm, and 15.7 ± 1.5 cm on days 9, 16, and 23, respectively); step widths narrowed (*P *= 0.002; 3.72 ± 0.40 cm, 3.45 ± 0.31 cm, 3.48 ± 0.39 cm on days 9, 16, and 23, respectively); and stride frequency did not change with time in all groups (*P *= 0.529; 2.65 ± 0.44 Hz, 2.69 ± 0.45 Hz, and 2.83 ± 0.56 Hz on days 9, 16, and 23, respectively). Despite the changes in percentage stance time balance and symmetry, differences in stride length, step width, and stride frequency were not observed between groups, even after correcting for the effects of velocity.

### Dynamic gait characteristics

**Figure 5:**
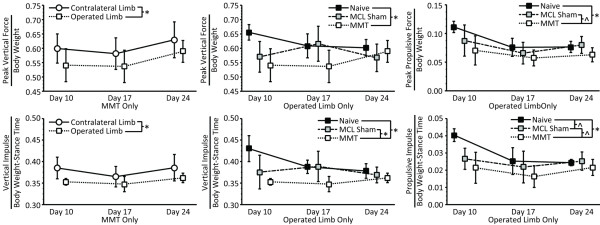
**Ground reaction forces**. Rats with medial meniscus transection (MMT) surgery had significantly different vertical force-time curves in their operated limb relative to their contralateral limb, as indicated by lower peak vertical force (top left, operated limb, open box; contralateral limb, open circle; *P *= 0.016) and vertical impulse (bottom left; operated limb, open box; contralateral limb, open circle; *P *= 0.003). Peak vertical force also varied between animals with MMT surgery and naive controls (solid box, *P *= 0.004), but not between animals with MMT surgery and MCL sham animals (shaded box, top middle). Vertical impulse, however, was lower in animals with MMT surgery relative to both naïve controls (*P *< 0.001) and medial collateral ligament (MCL) sham animals (*P *= 0.019, bottom middle). Like vertical force-time curves, braking-propulsion curves also varied between surgical groups, although changes were restricted to the propulsive phase of the curve. Peak propulsive forces were significantly lower in animals with MMT surgery relative to naïve controls (*P *= 0.003) and tended to be lower than those of MCL sham animals (*P *= 0.053, top right). Propulsive impulse was also significantly lower in animals with MMT surgery relative to naïve control (*P *< 0.001) and tended to be lower than MCL sham animals (*P *= 0.081, bottom right). Finally, MCL sham animals also tended to have a lower propulsive impulse than naïve controls (*P *= 0.079). Data are presented as mean ± standard deviation. *Differences between surgical groups that are significant at a level of *P *< 0.05; ^differences near significance at a level of 0.1 <*P *≤ 0.05.

Vertical ground reaction forces (Fz) differed significantly between surgical groups. Peak vertical force and vertical impulse of MMT rats were lower in the operated limb relative to the contralateral limb (*P *= 0.016; *P *= 0.003, respectively, Figure 5, left column). Operated limb peak vertical force and vertical impulse were also lower in MMT rats relative to naïve controls (*P *= 0.004; *P *< 0.001; respectively); in addition, operated limb vertical impulse was lower in MMT rats relative to MCL sham controls (*P *= 0.019; Figure 5, center column). Propulsive forces, but not braking forces, were altered in MMT rats. Peak propulsive force was significantly lower in MMT rats than in naïve control animals (*P *= 0.003) and tended to be lower than MCL shams (*P *= 0.053). Likewise, propulsive impulse was lower in MMT rats than in naïve controls (*P *< 0.001) and tended to be lower than MCL shams (*P *= 0.081; Figure 5, right column). MCL sham animals also tended to have lower propulsive impulses than naïve controls (*p *= 0.079). Mediolateral forces did not vary among treatment groups.

### Cytokine concentration

**Figure 6:**
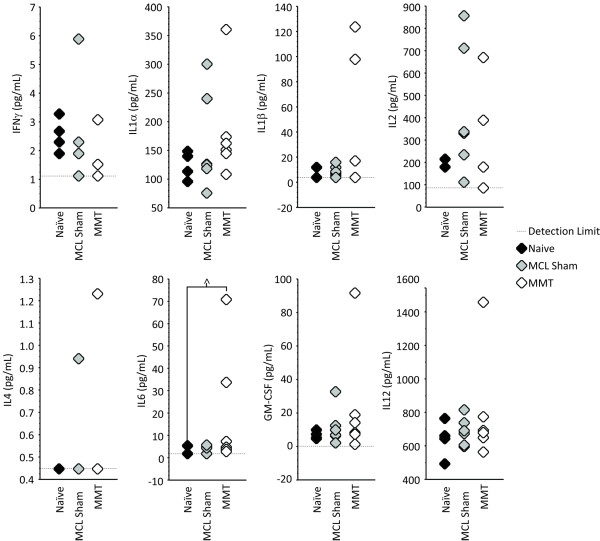
**Concentration of cytokines in the serum**. Although the concentration of cytokines in the synovial fluid after the 100-μl lavage was below detection limit for all cytokines in all samples, the concentrations of cytokines in sera were detectable. Significant differences among groups were not identified, although a general trend for higher concentrations of IL-1α, IL-1β, and GM-CSF in medial meniscus transection (MMT) rats may exist (nonsignificant). Moreover, serum concentration of IL-6 tended to be higher in rats with MMT surgery (open diamond) than that in naïve controls (solid diamond; *P *= 0.072), but not medial collateral ligament (MCL) sham animals (shaded diamond). Raw data are presented. ^Differences between surgical groups that are near-significant at a level of 0.1 <*P *≤ 0.05.

In sera, IL-1α, IL-12, and GM-CSF were detectable in all samples; IL-2, IL-6, and IFN-γ yielded relatively robust signals, detectable in 87.5%, 81.25%, and 75% of the samples, respectively; IL-1β and IL-4 were less robust, detectable in only 56.25% and 12.5% of the samples, respectively. IL-10 and TNF-α were below the detection limit in the sera from all animals. The concentration of cytokines in the synovial fluid was below the detectable limits in all samples, likely because of the use of a 100-μl lavage protocol that diluted all samples. Although significant differences among groups were not identified in the sera, IL-6 concentrations in the serum of MMT rats did tend to be higher than those of naïve controls (*P *= 0.072; Figure 6).

### Histology

Histology aligned well with past reports on the rat MMT model [12,31,32,44]. Rats with the MMT surgery had evidence of severe lesions on the medial tibial plateau (Table 1). These lesions graded between OARSI rank 4 (erosion) and OARSI rank 5 (denudation) [41] and ranked significantly higher than sections from the contralateral limb (*P *= 0.031), MCL sham animals (*P *= 0.005), and tended to rank higher than naïve controls (*P *= 0.068). Relative measurement of the lesion size, width, and depth revealed lesions in the operated limbs of MMT rats tended to be larger than those measured in the contralateral limb (*P *≤ 0.060) and were larger than those measured in MCL sham animals (*P *≤ 0.002) and naïve controls (*P *≤ 0.048).

**Table 1:** Histological grades and measurements

Surgical group	Knee	**OARSI Histopathology score (medial tibial plateau**, [41])	Lesion size (% of cartilage area)	Affected surface (% of cartilage surface)	Lesion depth (% of cartilage thickness)
Naïve	Left	1.5 ± 1.3	3.8 ± 3.8%	8.5 ± 6.3%	27.9 ± 47.8%

MCL sham	Left	0 ± 0	0 ± 0	0 ± 0	0 ± 0

MMT	Left	1.7 ± 2.1	5.2 ± 6.9%	12.7 ± 18.9%	36.2 ± 44.0%

Naïve	Right	0.5 ± 0.6	1.0 ± 1.4%	2.0 ± 2.6%	4.4 ± 6.2%

MCL sham	Right	0.2 ± 0.4	0.1 ± 0.3%	1.3 ± 3.0%	0.3 ± 0.8%

MMT	Right	4.5 ± 0.8^ab^	18.2 ± 6.5%^ab^	38.3 ± 15.8%^a^	85.5 ± 24.1%^a^

Rats with the medial meniscus transection (MMT) surgery had lesions that graded between OARSI rank 4 (erosion) and OARSI rank 5 (denudation) [41]; these lesions ranked significantly higher than histological sections from the contralateral limb (*P *= 0.031), medial collateral ligament (MCL) sham animals (*P *= 0.005), and tended to rank higher than naïve controls (*P *= 0.068). The size of lesion in the operated (right) limb, measured as the approximate percentage of cartilage loss, was larger in rats with MMT surgery than in rats with MCL sham surgery (*P *= 0.001) or naïve controls (*P *= 0.003); the size of the lesion in the operated limb of MMT rats also tended to be larger than that found in the contralateral limb control (*P *= 0.060). The percentage of the cartilage surface affected by the injury was larger in the operated limb of rats with MMT surgery than in contralateral limb controls (*P *< 0.001) and the limbs of rats with MCL sham surgery (*P *< 0.001) or naïve control animals (*P *< 0.001). Likewise, the percentage of the cartilage depth affected by the injury was larger in the operated limb of rats with MMT surgery than in contralateral limb controls (*P *< 0.010) and the limbs of rats with MCL sham surgery (*P *< 0.001) or naïve control animals (*P *< 0.007). Data are presented as mean ± standard deviation. ^a^Values that are significantly different at a level of *P *< 0.05; ^b^values near significance at a level of 0.1 <*P *≤ 0.05.

### Correlation analysis

Limb sensitivity, gait symmetry, percentage stance time balance, peak vertical force, vertical impulse, peak propulsive force, propulsive impulse, and serum IL-6 concentration obtained at 24 days were studied for potential correlations with measures of cartilage remodeling in the operated (right) knee (Table 2), including OARSI score on the medial tibial plateau, and lesion size reported as percentage cartilage loss, surface loss, or depth loss. Despite evidence of differences in each of these parameters among treatment groups, only percentage stance time balance, gait symmetry, peak propulsive force, and serum IL-6 concentration demonstrated significant correlative associations with measures of cartilage remodeling and lesion formation (*P *< 0.05). Of these, serum IL-6 concentration had the strongest correlative association with percentage cartilage loss, as well as with percentage surface, depth loss, and OARSI score. Percentage stance time balance, peak propulsive force, and gait symmetry were similarly found to associate with some, but not all, of these histological parameters.

**Table 2:** Correlations between 28-day measures and cartilage lesion size

	OARSI score (medial tibial plateau)	Lesion size (% of cartilage loss)	Lesion size (% of surface affected)	Lesion size (% of depth affected)
Operated limb sensitivity (mech. allodynia)	0.0084	0.0155	0.0054	0.0191
	-0.020 ± 0.059	-0.001 ± 0.003	-0.002 ± 0.006	-0.006 ± 0.012
	0.736	0.646	0.787	0.611

Gait symmetry (spatiotemporal gait)	0.1371	0.1439	**0.2851**	0.1587
	38.4 ± 25.7	1.74 ± 1.14	**5.22 ± 2.21**	8.36 ± 5.14
	0.158	0.148	**0.034^a^**	0.127

Perc. stance time balance (spatiotemporal gait)	**0.3526**	**0.3443**	*0.2178*	**0.3505**
	**67.2 ± 24.3**	**2.94 ± 1.08**	*4.98 ± 2.52*	**13.6 ± 4.9**
	**0.017^a^**	**0.017^a^**	*0.069*^b^	**0.016^a^**

Peak vertical force (dynamics, dimensionless)	0.0000	0.0009	0.0003	0.002
	-0.12 ± 14.47	-0.07 ± 0.64	0.09 ± 1.37	-0.14 ± 2.93
	0.994	0.911	0.947	0.963

Vertical impulse (dynamics, dimensionless)	0.1042	0.1023	0.0341	0.0874
	-44.6 ± 34.9	-1.95 ± 1.55	-2.40 ± 1.26	-8.25 ± 7.13
	0.223	0.228	0.494	0.267

Peak propulsive force (dynamics, dimensionless)	*0.2016*	**0.2804**	**0.3464**	0.1757
	*-68.6 ± 36.5*	**-3.58 ± 1.53**	**-8.49 ± 3.11**	-13.0 ± 7.5
	*0.082*^b^	**0.035^a^**	**0.017^a^**	0.106

Propulsive impulse (dynamics, dimensionless)	0.0797	0.1355	0.1758	0.0718
	-137 ± 125	-7.92 ± 5.35	19.2 ± 11.1	-26.3 ± 25.3
	0.290	0.161	0.106	0.316

Serum IL-6 concentration (log transformed)	**0.3120**	**0.5085**	**0.2694**	**0.2778**
	**1.13 ± 0.45**	**0.064 ± 0.017**	**0.099 ± 0.043**	**0.22 ± 0.09**
	**0.025^a^**	**0.002^a^**	**0.040^a^**	**0.036^a^**

Outcome measures of animal behavior or serum cytokine concentrations that gave evidence of significant (p < 0.05) or near-significant differences among groups (0.05 ≤ p < 0.10) were investigated for correlation to measures of cartilage remodeling and lesion formation. Standard linear regressions were fit to the data set with either OARSI score on the medial tibial plateau or lesion size measured by percentage of cartilage loss, surface loss, or depth loss as the dependent variable (columns). All outcome measures of animal behavior and serum cytokines were obtained at 28 days. Data are presented as goodness of fit (R^2^, top), the slope coefficient (β_1_, middle, mean ± standard deviation), and significance of the slope coefficient (p value of β_1_, bottom). A significant slope coefficient indicates that the measure of animal behavior or serum cytokine concentration associates with the measure of cartilage remodeling and lesion formation at 28 days. **^a^**Significant correlations are presented in bold; ^b^near-significant correlations are in italics.

## Discussion

To our knowledge, this study is the first description of functional losses after medial meniscus injury in the rat with reported changes in gait dynamics. After meniscal injury, rats walked with imbalanced gaits that became progressively asymmetric with time, indicating that rats with meniscal injury spent less time on their injured limb, with a slight limp developing over time. Similarly, ground reaction force differences provided evidence of meniscal injury. Peak vertical force and vertical impulse were lowest in the MMT group, confirming that animals with medial meniscus injury bore significantly less load on their operated limb during walking. Similar to vertical forces, peak propulsive force and propulsive impulse reduced after medial meniscus injury. In general, gait data aligned well with the previously reported behavioral measures for tactile allodynia and incapacitance meter-based weight bearing [12,31], indicating that behavioral assessments can describe functional and symptomatic consequences of knee instability in the rat.

The data reported herein indicate that gait analyses are sensitive to knee joint instability and cartilage remodeling. In particular, stance time imbalance, vertical impulse, and peak vertical force yielded more robust comparisons of the affected and contralateral limb than weight-bearing, as measured by an incapacitance meter. Moreover, gait symmetry demonstrates a general trend toward increased asymmetry as OA progresses; no other parameter, including tactile allodynia and weight-bearing, demonstrated this potential. Possible explanations for the increased sensitivity of gait recordings relative to tactile allodynia and incapacitance meter tests are the multidimensional and quantifiable nature of gait recordings, the sensitivity of the high-speed equipment, and a comparatively stimulus-free environment that may limit stress-induced responses in the animal. With end-point data, gait symmetry, percentage stance time balance, and peak propulsive force were found to have a significant correlative association with the size of cartilage lesions at 24 to 28 days. These observed associations were of modest magnitude but statistically significant, describing between 20% and 50% of the variation observed in cartilage remodeling.

Although peak vertical force and vertical impulse demonstrated significant differences between the affected and contralateral limbs, the associations between these measures and OA histopathology were poor. Body weight is supported by all four limbs of the quadruped; thus, it is possible that vertical force and impulse can be supported by the forelimbs through the use of gait patterns that shift an animal's center of mass toward the forelimbs. However, propulsive forces are much larger in a rat's hindlimbs than its forelimbs during locomotion; thus, propulsive force measures may be more sensitive to hindlimb injury. Moreover, in our data, hindlimb peak propulsive force demonstrated correlations to the severity of knee OA at 24 to 28 days, whereas vertical force and vertical impulse did not.

Although significant differences between surgical groups were not observed for serum concentrations of IL-6 (*P *= 0.072; Figure 6), the strongest correlation between cartilage remodeling and an end-point measure was found for serum concentrations of IL-6. It is unfortunate that the concentration of cytokines in the synovial fluid was not possible to obtain in the MMT model by using a 100-μL lavage. It is probable that the lavage volume diluted the cytokine concentration in the synovial fluid below that which can be detected by using the Rat Cytokine 10-Plex Panel. Other techniques, such as absorbing synovial fluid to filter paper or an alginate sponge [45], may yield better results for the recovery of synovial fluid in the MMT model in the future. Although the manufacture states that the assay is compatible with buffered solutions, it is also possible that the 10-Plex Panel is not capable of measuring cytokine concentrations in the synovial fluid. Nonetheless, the data reported herein support a multifaceted approach spanning both behavioral and biomarker analyses. Like many biomarker assays, behavioral characteristics can be acquired longitudinally in the same research animal (urine, saliva, and serum), and the combination of biomarker and behavioral data may prove powerful in providing a non-invasive associative or predictive relation of joint-level cartilage damage in the preclinical model.

It should be noted that the associations described herein are constructed from a limited cohort of 18 animals, and thus, larger cohorts and longitudinal assessments across a range of OA severity are necessary to construct fully an associative or predictive relation. However, knowing from prior work that cartilage damage increases in severity over time in the MMT model [32], we describe behavioral data that has a potential association with changing OA severity. Gait symmetry appears particularly promising for associating with increasing cartilage damage. Most gait parameters and mechanical sensitivity had sizable shifts at day 9 that either maintained or decreased in magnitude over time. Gait symmetry, however, demonstrated a temporal shift, with gait becoming progressively asymmetric over time. This observation suggests that many gait parameters and mechanical sensitivities are shifted because of the meniscal tear surgery, whereas gait asymmetry may result from the joint remodeling that follows the meniscal injury.

The gait analysis techniques used for this report require manual digitization of foot-strike and toe-off events; this process is laborious and time consuming. Commercial systems that automate the collection of gait events are available [34,46-55]; however, many of these systems currently record at 100 frames per second. Even with idealized digitization codes, these systems in their current configuration are unlikely to be sensitive enough to detect stance time imbalance and gait asymmetry shifts between 1% and 3%. Other limitations of gait testing are the need to manage large data sets and to account for covariates such as velocity and weight. Although we selected manual digitization to optimize recording speeds for the small gait compensations observed in the MMT model, continued development of automated gait systems will help to increase the throughput and decrease the labor costs currently associated with gait analyses [21-23,50,56-58].

It also should be noted that prior studies have reported larger differences on the incapacitance meter than those reported herein [12,31]. A potential explanation for this discrepancy is simply the analysis method used. Bove and co-workers [12] found a significant shift in weight distribution on an incapacitance meter by using subtraction of left-foot weight from right-foot weight with eight animals in each treatment group. Here, weight distribution was evaluated by using a normalization method that is less biased by changes in total body weight, but with only six animals per treatment group. In another relevant study, Fernihough and co-workers [31] also failed to identify imbalanced weight distribution on an incapacitance meter after meniscal injury in the rat, although a trend toward decreased weight bearing on the operated limb can be reasoned from their data set. Despite inconsistencies among these studies, the combined results from these reports likely indicate that weight distribution is imbalanced after meniscal injury in the rat. Indeed, the gait data reported herein support the conclusion that animals with MMT surgery were compensating for the operated right limb with the contralateral left limb through both changes in vertical impulse and percentage stance time balance. Weight distribution imbalance on an incapacitance meter may have been indicated with more animals; however, by using the same animal number, gait analysis revealed compensations in the affected limb. The combined results from our study and related work in the literature [12,31] indicate that behavioral shifts in the MMT model are subtle, and highly sensitive and repeatable methods are necessary to identify these changes. Moreover, the data reported herein suggest that gait analyses of rodent walking may be more sensitive to changes caused by meniscal instability than by weight bearing as assessed by an incapacitance meter and may improve an ability to detect differences between the operated and contralateral limb.

Similar gait disturbances have also been described in a rat anterior cruciate ligament (ACL) transection model of OA [55]. Here, ACL transection reduced peak vertical force in the affected limb relative to the contralateral limb. Intra-articular injection of lubricin, a molecule that plays a central role in joint lubrication and synovial homeostasis [59,60], reduced hindlimb peak vertical force ratios; however, animals treated with lubricin loaded their ACL-deficient knee more than their contralateral knee. Whereas the TekScan system used to analyze the gait of rats after ACL transection can acquire paw pressures across multiple strides [55], our system is capable of recording three-component ground reaction forces in time at 200 Hz or faster. Both systems have distinct advantages and disadvantages; but, this prior work and the data described herein clearly identify the ability to detect gait changes in rat models of unilateral knee OA and the utility of these measures in testing potential therapeutics.

MCL transection alone did not yield significant changes to rodent gait relative to naïve controls by 24 days after operation, although propulsive impulse did tend to decrease with MCL transection. Loss of the MCL should result in a valgus instability [61], and more-sophisticated gait analyses such as inverse dynamics may reveal more detail for changes in loading that result from MCL transection alone [62]. However, these analyses are difficult to accomplish in the rat because of large inaccuracies that occur between skin markers and true joint position in rodents [63]. Furthermore, longer time points could eventually result in the divergence of the gait characteristics for MCL sham and naïve animals, as loss of the MCL may eventually lead to degenerative changes in the joint's articulating surfaces.

It should be noted that our data describe gait compensations indicative of a unilateral injury, where stance times are imbalanced, the gait pattern is asymmetric, and vertical force (limb loading) is shifted to the contralateral limb. For bilateral injuries, these compensations may not occur, because an uninjured contralateral limb is not available to support the affected limb. For bilateral injuries, stance times will likely remain balanced and symmetric if injuries are comparable between limbs; however, selected walking velocities tend to decrease, and shorter, more-frequent strides are used at a given velocity. This gait pattern effectively reduces the amount of time a limb must support weight on its own (single-limb support phase). Moreover, we previously observed compensations indicative of bilateral injury in a genetic knockout mouse [20]; thus, gait analysis can be used to track both the unilateral and bilateral injuries in the rodent preclinical model.

## Conclusions

Our results indicate that rodent gait characteristics, both spatiotemporal analysis of the gait pattern and dynamic analysis of ground reaction forces, are capable of tracking the symptomatic consequences of knee instability in the rodent model. Moreover, as gait compensations observed in the MMT animals were significantly larger than those observed in animals with MCL injury alone, our data indicate that gait compensations in the MMT model are most likely due to meniscal instability and articular cartilage damage, not MCL transection alone. Some characteristics, such as an asymmetric gait pattern, may develop as the severity of cartilage lesions progresses. Although additional work is required to understand fully and to verify the relation between these parameters and cartilage lesion formation, our data describe the potential of a behavioral measure to track directly or to associate with cartilage lesion formation in a rodent OA model; moreover, these noninvasive gait measures provide functional assessments of OA in animal models that have some validity to functional compensations observed in humans.

## Abbreviations

ACL: Anterior cruciate ligament; ANOVA: analysis of variance; GLM: generalized linear model; GM-CSF: granulocyte-macrophage colony-stimulating factor; IL-1α: interleukin-1α; IL-1β: interleukin-1β; IL-2: interleukin-2; IL-4: interleukin-4; IL-6: interleukin-6; IL-10: interleukin-10; IL-12: interleukin-12; INF-γ: interferon-gamma; MMT: medial meniscus transection; MCL: medial collateral ligament; NAD: nicotinamide adenine dinucleotide; NIAMS: National Institute of Arthritis and Musculoskeletal and Skin Disease; OA: osteoarthritis; OARSI: Osteoarthritis Research Society International; TNF-α: tumor necrosis factor-alpha; Tukey HSD: Tukey honest significant difference.

## Competing interests

The authors declare that they have no competing interests.

## Authors' contributions

KDA conducted all behavioral analyses and drafted the manuscript. BAM conducted the surgical procedures with the surgical assistance of MAG; SBA provided surgical training on the MMT surgery to both BAM and MAG before the study. BAM, SBA, MAG, and KDA performed the dissections and obtained the sera and synovial fluid. MAG and KDA sectioned the histological samples, and KDA and BAM graded the sections. JLH and VBK conducted the sera and synovial fluid analysis for cytokine concentration. DOS provided the force-plate equipment and assisted KDA in the interpretation of the ground reaction force data. LAS supervised all stages of the experiment and assisted in the interpretation and analysis of the data. KDA and LAS obtained the funding for the experiment. All authors read and approved the final manuscript as presented.
